# How to Treat Type B Aortic Dissections in the Presence of an Aberrant Right Subclavian Artery: A Systematic Review

**DOI:** 10.1055/s-0042-1757948

**Published:** 2023-02-27

**Authors:** Francesco Lombardi, Apostolos Mamopoulos, Jaroslav Benedik, Marcus Katoh, Knut Kröger, Gabor Gäbel

**Affiliations:** 1 Department of Vascular Surgery, Helios Clinic, Krefeld, Germany; 2 Department of Cardiac Surgery, Helios Clinic, Krefeld, Germany; 3 Department of Diagnostic and Interventional Radiology, Helios Clinic, Krefeld, Germany; 4 Department of Vascular Medicine, Helios Clinic, Krefeld, Germany

**Keywords:** aberrant right subclavian artery, arteria lusoria, Type B aortic dissection

## Abstract

An aberrant right subclavian artery (ARSA) is the most common congenital variant of the aortic arch. Usually, this variation is largely asymptomatic, but sometimes it may be involved in aortic dissection (AD). Surgical management of this condition is challenging. The therapeutic options have been enriched in recent decades by establishing individualized endovascular or hybrid procedures. Whether these less invasive approaches bear advantages, and how they have changed the treatment of this rare pathology, is still unclear. Therefore, we conducted a systematic review. We performed a review of literature from the past 20 years (from January 2000 until February 2021) complying with the Preferred Reporting Items for Systematic Reviews and Meta-analyses guidelines. All reported patients treated for Type B AD in the presence of an ARSA were identified and classified into three groups according to the received therapy (open, hybrid, and total endovascular). Patient characteristics, as well as in-hospital mortality, and major and minor complications were determined and statistically analyzed. We identified 32 relevant publications comprising 85 patients. Open arch repair has been offered to younger patients, but significantly less often in symptomatic patients needing urgent repair. Therefore, the maximum aortic diameter was also significantly larger in the open repair group compared with that in the hybrid or total endovascular repair group. Regarding the endpoints, we did not find significant differences. The literature review revealed that open surgical therapies are preferred in patients presenting with chronic dissections and larger aortic diameters, most likely because they are unsuitable for endovascular aortic repair. Hybrid and total endovascular approaches are more often applied in emergency situations, where aortic diameters remain smaller. All therapies demonstrated good, early, and midterm outcomes. But, these therapies carry potential risks in the long term. Therefore, long-term follow-up data are urgently needed to validate that these therapies are sustainable.

## Introduction

An aberrant right subclavian artery (ARSA)—or arteria lusoria—is the most common congenital variant of the aortic arch and may occur in 0.5 to 1.8% of the population. 1 This variation is largely asymptomatic and often an incidental finding. Due to its course, the aberrant artery may predispose to esophageal compression, known as dysphagia lusoria, or cause cough and airway obstruction. The origin of the ARSA may undergo aneurysmal dilatation, producing the entity known as Kommerell's diverticulum. In these cases, compressive symptoms are more likely and there is also a risk of embolization and rupture. 2 Much less frequently, this rare variation is involved in aortic dissections (ADs), either as the site of the primary intimal tear or as a dissected aortic branch. Whereas asymptomatic patients with stable Type B AD do not need surgical therapy, symptomatic patients or those with progressive aortic diameter need an enduring repair. In cases of malperfusion, the aim of the therapy is to minimize the ischemia time of affected organs as soon as possible.

**Fig. 1:**
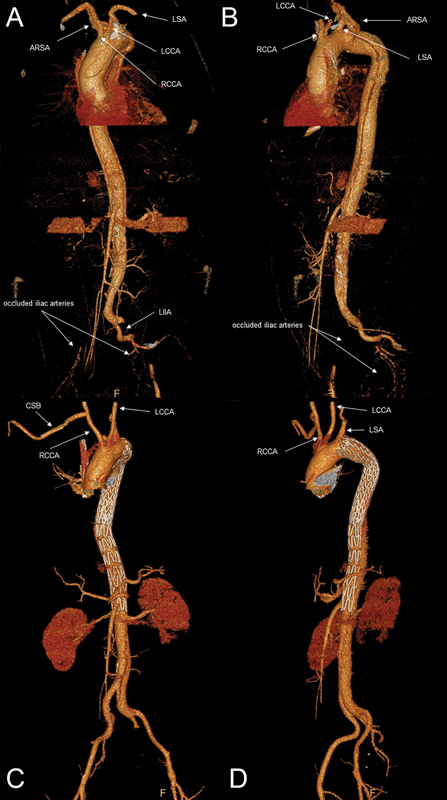
Pre- and postoperative computed tomography (CT)-angiography of a 49-year-old patient. Initial CT demonstrated a Type B aortic dissection (AD) originating just distal to the take-off of the left subclavian artery and extending into both common iliac arteries **(A, B)** . Imaging revealed the presence of a dissected aberrant right subclavian artery in this bovine-type arch, where the left common carotid originates at the same level as the right does. The right vertebral artery was involved in the dissection. Maximum aortic diameter was determined at 44 mm in the descending thoracic aorta. There was an imminent true lumen collapse at the level of the visceral vessels. The dissection involved the origin of the superior mesenteric artery. The right renal artery originated from the true lumen, whereas the left renal artery originated from the false. The dissection continued into both common iliac arteries, causing an occlusion of both external iliac and femoral arteries. Under general anesthesia, a hybrid approach was conducted. A right common carotid artery to right subclavian artery bypass was implanted (8 mm Hemaguard UT, Maquet Getinge Group GmbH; Rastatt, Germany). Immediately afterward, a Gore cTAG thoracic aorta stent graft (TGM373720E; W.L. Gore & Associates Inc., Flagstaff, AZ) was deployed just distal to the LSA, obliterating flow into the false lumen and covering the ostium of the ARSA. Since the true lumen stayed compromised in the visceral segment of the aorta, we decided to optimize peripheral perfusion by additionally implanting a noncovered Zenith endovascular stent (ZDES-36-180; Cook Medical, Bloomington, IN). The follow up CT scan 5 months afterward showed normal perfusion of all aortic branches and a significant remodeling of the thoraco abdominal aorta **(C, D)** . At this time point, the patient has recovered completely. ARSA, aberrant right subclavian artery; CSB, carotid artery to right subclavian artery bypass; LCCA, left common carotid artery; LSA, left subclavian artery; RCCA, right common carotid artery.

Recently, in our clinic, such a patient presented with malperfusion of both legs and an imminent aortic true lumen collapse at the level of the visceral vessels ( Fig. 1 ). Even though a variety of approaches have been described in the literature, decision-making has to be quick in such a situation. Due to advancements in endovascular techniques during the last decades, the so-called hybrid or total endovascular procedures are increasingly applied to treat Type B AD. 3 Unfortunately, in the presence of congenital variants of the arch, an endovascular approach is much more challenging and the outcome may be jeopardized. Since there are currently no recommendations for the treatment of Type B AD in the presence of an ARSA, we performed a selective literature review to analyze the therapeutic results for this pathology. Several questions were addressed as follows:

- Which therapies are currently most often applied? Is there an emerging trend?
- Do the results of these therapies differ?
- Are there specific characteristics or circumstances that indicate a specific therapy?
- Are there specific complications of the different approaches of which we should be aware of?

## Materials and Methods

This review was performed in accordance with the Preferred Reporting Items for Systematic Reviews and Meta-analyses (PRISMA) guidelines for systematic reviews and according to a defined protocol registered with PROSPERO (York University, York, United Kingdom) prior to commencing the review ( Supplementary Appendix ).

### Identification of Studies

PubMed and Medline databases were searched by two authors (F.L. and G.G.) for articles published from January 1, 2000, until February 1, 2021. The reference lists of all eligible studies and relevant narrative reviews were also hand searched. Accordingly, the two authors performed a targeted search of the literature, including abstracts published in major cardiovascular and pathology journals. The detailed search syntax and search string are available in Supplementary Material S1 .

### Inclusion/Exclusion Criteria

All prospective and retrospective studies, as well as all case reports and series, providing information about the clinical manifestation and management of patients diagnosed with Type B AD in the presence of an ARSA were considered. We excluded all cases in which the AD affected the ascending aorta. Descriptive radiological reports, as well as commentaries, were also excluded. Where multiple reports from the same center and author were identified that resulted in duplication of cases, the first reporting study was included.

### Article Selection

Two reviewers independently assessed each title and abstract of all identified citations. Full-text articles were obtained if either reviewer considered the citation relevant, with a low threshold for retrieval. Full texts were then reviewed critically to assess eligibility. Finally, each record was reviewed by the authors and all reported cases with a dissection starting at the level of the left subclavian artery (LSA) or beyond, and in the presence of an ARSA, who received the treatment were included in the analysis. Reasons for exclusion were recorded ( Supplementary Material S2 ). It was decided not to use any risk of the bias assessment tool. It was anticipated that all records would probably be observational in nature. No study was excluded based on methodology alone.

### Data Extraction

Data collection from all included studies was performed independently and in duplicate. For each record, we retrieved information in a predefined Microsoft Excel spreadsheet, including the number of patients and their characteristics, symptoms, treatment modalities, complications, and follow-up evaluations including reinterventions ( Supplementary Materials S3 – S5 ).

### Outcomes

We classified the patients into the following three groups of therapies:

- Open group: open surgical approaches like total arch repair (TAR) and elephant trunk technique (conventional or frozen)—all via thoracotomy or median sternotomy using extracorporeal perfusion.
- Hybrid group: combined procedures with open extra-anatomic bypass or debranching surgery and thoracic endovascular aortic repair (TEVAR) without cardiopulmonary bypass.
- Total endovascular group: endovascular arch repair by TEVAR with and without chimney/periscope technique or fenestrated endoprosthesis.

All available patient characteristics, as well as periprocedural or follow-up complications, were assessed. We determined if the groups demonstrated statistically significant differences regarding their patient characteristics, and we recorded the following endpoints: in-hospital mortality; major complications (including in-hospital mortality and stroke); minor complications (postprocedural bleeding, endoleaks, bypass occlusions, retrograde Type A AD, and myocardial infarction); and follow-up complications (secondary thoracoabdominal aortic aneurysm, stroke, penetrating aortic ulcer, and aortoesophageal fistula).

### Statistical Analysis

Demographic patient data and results are presented as mean ± standard deviation or as absolute and relative frequency. A comparison of categorical data between treatment groups was performed using the Fisher's exact test. The Mann–Whitney *U* -test was performed to identify differences in continuous variables. One-way analysis of variance (Kruskal–Wallis test) was performed for group analysis. A *p* -value <0.05 was considered statistically significant. All statistical analyses were performed using the Graphpad Prism 6.0 statistical package (Graph Pad, La Jolla, CA).

## Results

### Characteristics of Included Reports

**Fig. 2:**
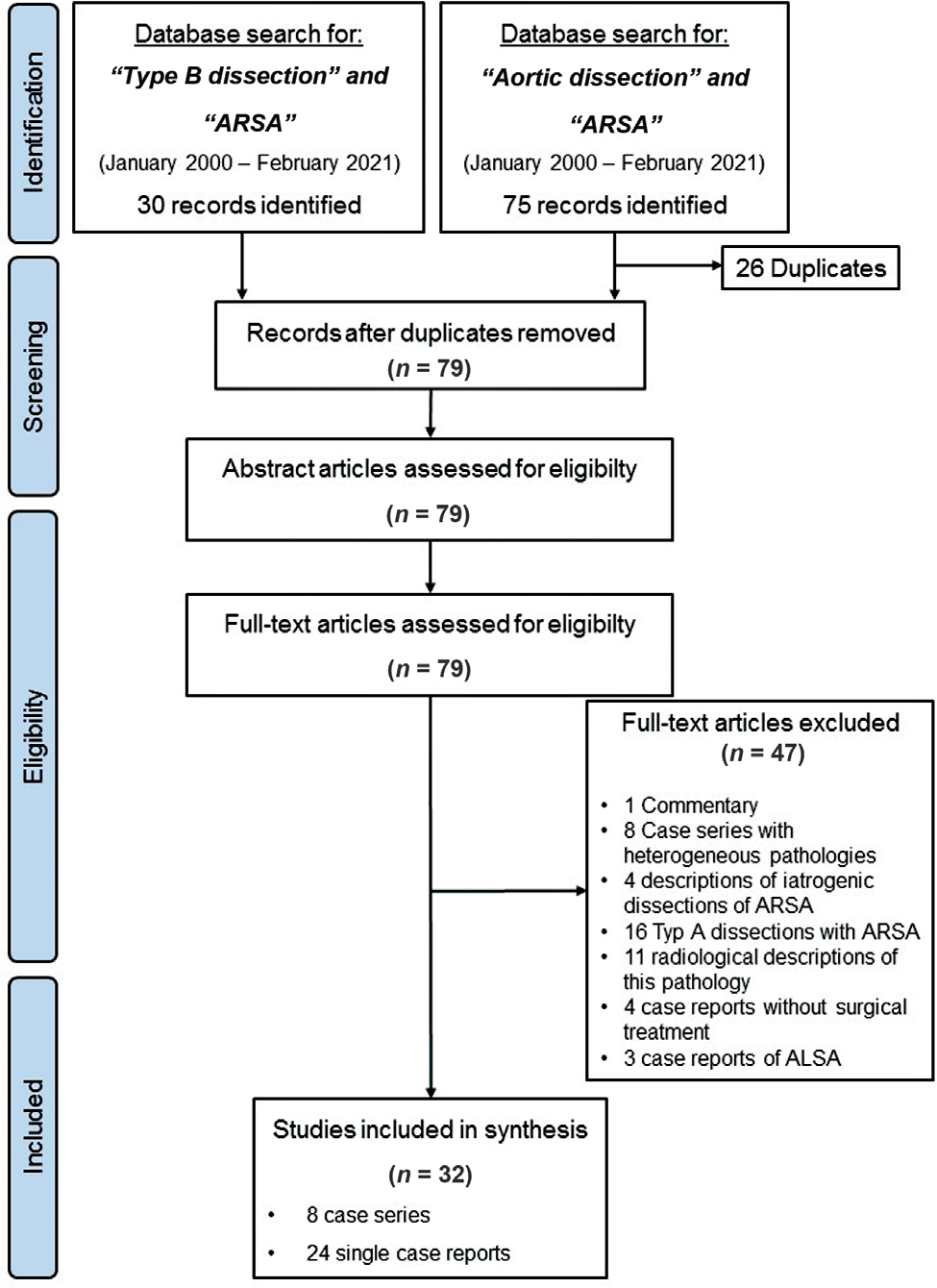
Preferred Reporting Items for Systematic reviews and Meta-analysis flow diagram for literature search to identify reports of treatment modalities for patients presenting with Type B aortic dissection in the presence of an aberrant right subclavian artery.

The final review included eight relevant case series 4 5 6 7 8 9 10 11 and 24 single case reports 12 13 14 15 16 17 18 19 20 21 22 23 24 25 26 27 28 29 30 31 32 33 34 35 comprising a total of 85 patients ( Fig. 2 ). The majority of cases have been reported during the past 10 years (90.7% of patients after 2010). We did not observe a general trend toward any therapeutic approach over time. All reports originated from larger centers with high expertise. Most cases were reported from China ( *n* = 61; 71.2%). In each treatment group, always the largest case series originated from a Chinese high-volume center where up to 16 patients with Type B AD in the presence of an ARSA presented within 5 years. 8

### Treatment Modalities

We identified significant differences within patient characteristics for the approaches ( Table 1 ). Open surgical repair was offered to significantly younger (predominantly male) patients and significantly less often to symptomatic patients needing urgent repair. The maximum aortic diameter was also significantly larger in the open repair group compared with that in the hybrid or total endovascular repair group.

**Table 1:** Results of the literature review regarding the three different therapeutic approaches

	All	Open repair	Hybrid repair	Total endovascular repair	*p* -Value
*n*	85	22	25	38	NS
Male gender (%)	77.6	90.9	68.0	76.3	NS
Age (y)	53.9 ± 10.4	49.1 ± 8.7	52.1 ± 10.6	57.8 ± 9.9	**0.0051**
Symptomatic dissection (%)	62.8	50.0	95.8	86.7	**0.0007**
Maximum aortic diameter (mm)	42 ± 9.6	50 ± 8.5	40 ± 9.1	39 ± 8.5	**0.0001**
In-hospital mortality (%)	2.4	4.5	0.0	2.7	NS
Major complications (%)	6.0	13.6	0.0	5.4	NS
Minor complications (%)	23.5	28.6	28.0	16.2	NS
Follow-up complications (%)	15.8	5.9	20.8	17.1	NS
Follow-up (month)	25 ± 29	28 ± 41	23 ± 12	29 ± 14	NS

Abbreviation: NS, not statistically significant.

Regarding the endpoints, we did not find significant differences. Especially, when looking at reported in-hospital mortality, with only two deaths reported, there were no relevant differences. One in-hospital death occurred in the open repair and one in the total endovascular group. The patient from the endovascular group had to be converted to an open repair after an endovascular repair failed technically. 7 10 Both patients died in the postoperative course due to multiorgan failure.

Regarding the incidence of minor complications, we observed no significant differences. But, we observed differences in the type of complication, for example bleeding, compression of the trachea, and critical illness polyneuropathy in the open group versus endoleaks and right arm ischemia in the hybrid and total endovascular group ( Table 2 ).

**Table 2:** Overview of results for different approaches described in current case series/reports

Intervention		Number of patients	In-hospital-mortality	Major complications	Minor complications	Number of reintervention	Follow-up (mo)
Open	TAR + TEVAR	8	1	1	0	0	31
	Frozen elephant trunk (FET)	8	0	0	Three (slow weaning, compression trachea, bleeding)	One (bleeding)	9.6
	TAR	5	0	1	Three (critical illness polyneuropathy, paresis, wound infection)	Two (for TAAA, pectoralis flap)	50.7
	Distal arch repair	1	0	0	0	0	0
Hybrid	TEVAR + bypass/debranching only RSA	6	0	0	Three (SMA stenose, endoleak type Ia, plexus injury)	One (PTA SMA)	16.8
	TEVAR + bypass LSA and coiling RSA	2	0	0	0	0	6.5
	TEVAR + bypass LSA and RSA	10	0	0	Two (endoleak type Ia, plexus injury)	0	34.6
	TEVAR + Bypass RCCA-RSA-LCCA-LSA	5	0	0	Two (endoleak type Ia, and type II)	One (coiling type II endoleak)	18
	TEVAR + ascendocarotid subclavian bypasses	2	0	0	0	0	13.5
Total endo	TEVAR + chimney/snorkel (periscope)	17	1	1	Two (endoleak type Ia, and right arm ischemia)	0	28.8
	fenestrated/branched TEVAR	2	0	0	0	0	10
	TEVAR	19	0	1	Four (endoleak type Ia, and three transient right arm ischemia)	Two (both type Ib endoleaks)	28.9

Abbreviations: LCCA, left common carotid artery; LSA, left subclavian artery; PTA, percutaneous transfemoral angioplasty; RCCA, right common carotid artery; RSA, right subclavian artery; SMA, superior mesenteric artery; TAAA, thoracoabdominal aortic aneurysm; TAR, total arch repair; TEVAR, thoracic endovascular aortic repair.

In the hybrid group, a variety of prosthetic bypasses to the supra-aortic branches were performed. Only one transposition—right subclavian artery (RSA) into right common carotid artery (RCCA)—was documented. 33 Transient brachial plexus lesions were reported in two patients, both resolving during follow-up. We did not identify any significant differences regarding the endpoints for the different approaches to revascularize the supra-aortic vessels. The same applies for the total endovascular group.

In the total endovascular group, 19 patients were treated by TEVAR only. In seven of these cases, the ostium of the ARSA was preserved and not completely covered by the stent graft. In the other 12 cases, the ARSA was covered and three patients (25%) suffered reversible claudication of the right arm. 9 10 16 Interestingly, in the case series by Zhou et al, 10 two patients were initially treated by TEVAR with coverage of the ARSA and received a periscope stent after developing signs of right arm ischemia.

### Follow-up

Only few follow-up complications were documented in the analyzed literature. In the open surgery group, one patient, who received a TAR, developed a thoracoabdominal aneurysm and required aortic replacement during follow-up. 6 Complications during follow-up in the hybrid group were two asymptomatic bypass occlusions, a type II endoleak needing reintervention, and a retrograde Type A AD (3 months after TEVAR) in one patient with an initial endoleak type Ia, who refused further therapy and was closely monitored. 8 In the total endovascular group, one patient developed a penetrating aortic ulcer distal to the stent graft and another patient developed a type Ib endoleak. Both patients received distal extensions. One patient developed an aortoesophageal fistula with lethal course, 17 and one minor stroke without permanent disability was observed in another patient.

## Discussion

The coexistence of Type B AD and ARSA is rare. There is common agreement that a diseased ARSA (aneurysmal or involved in an AD) carries a higher risk for a negative outcome. 36 However, due to the lack of larger studies, no standard surgical approach has yet been established. For the first time, we reviewed the available literature on patients treated for Type B AD in the presence of an ARSA. We categorized the patients in three treatment groups. No significant differences were identified regarding in-hospital mortality or major or minor complications. In general, all three approaches demonstrated satisfactory short- and midterm results which are in accordance with a similar analysis of ARSA repair without AD. 37 38

Especially, the reported mortality (4.5%) for open surgical reconstructions in the presence of an ARSA with the need of cardiopulmonary bypass has improved over recent decades. Since the first successful surgical treatment of a symptomatic ARSA, 75 years of experience have been accumulated with open surgical treatment of ARSA. 39 Various techniques for aortic repair and reconstruction of ARSA have been described, with varying mortality rates. In 1994, Kieffer et al 36 published their 16-year single-center experience, including 33 patients, with the surgical therapy of arch pathologies with ARSA. Their results were generally satisfactory, although four patients died (12.1%) in the early postoperative phase. Later series regarding open surgery have reported similar mortality rates, between 9 and 18%. 40 The establishment of the conventional elephant trunk and frozen elephant trunk technique has further reduced mortality rates in patients treated for ARSA pathologies. 38 Our analysis of the available literature for Type B AD in the presence of ARSA has confirmed this trend. The main advantage of the elephant trunk technique is immediate thrombosis and secondary remodeling of the dissected descending aorta, reducing the risk for secondary dilatation. 41 This is a relevant issue since, in the group of patients who received only an open arch repair, one patient suffered secondary dilatation and needed reintervention during follow-up. Nevertheless, despite advances in surgical and perioperative management, open procedures remain demanding and invasive. Thus, patients deemed unfit or patients who refuse open surgery may be offered a less invasive approach. In general, our analysis revealed that today, open surgery seems to be reserved for patients unsuitable for endovascular approaches, usually because their aortic diameter has enlarged to the point of jeopardizing adequate sealing. This explains why there are significantly more asymptomatic patients in the open surgery group, where the indication for therapy is secondary dilatation of the dissected aorta.

Since the hybrid and total endovascular approaches do not need a cardiopulmonary bypass, these therapies are considered less invasive. Another advantage of these procedures is that they are usually quicker and patients usually recover much faster. Unfortunately, we were unable to compare the duration of hospital stay for the different approaches, since only few publications reported this.

There are few considerable prerequisites for hybrid or total endovascular repair for Type B ADs in the presence of an ARSA: sufficient size, as well as limited tortuosity, of the access arteries, and suitable proximal and distal landing zones for the stent graft. Tortuosity seems not highly relevant in these predominantly young patients. Also, distal landing zones are not as important in dissected aortas, since remodeling will lead to shrinkage and eventual alignment of the distal aortic wall to the stent graft. Most relevant is an adequate proximal landing zone. This landing zone is most often proximal to the origin of the ARSA and sometimes even to the LSA. Our review demonstrates that one can achieve sufficient sealing in many different ways: TEVAR over the ostium and coil or plug embolization of the ARSA; TEVAR with debranching or extra-anatomic bypassing of supra-aortic vessels, TEVAR with chimney or periscope stenting of the ARSA and LSA, or application of fenestrated stent grafts for the aortic arch.

In the current literature, some urgent cases are described in which only a TEVAR was performed without revascularization of the RSA. This approach may jeopardize perfusion of the right upper extremity as well cerebral perfusion. In one series, this approach was performed in eight patients, two of whom did suffer symptomatic ischemia of the right arm. Therefore, the team decided to perform periscope stenting to maintain flow to the right upper extremity. 10 In the current largest report on total endovascular repair from Zhang et al, 9 six patients received TEVAR without additional endoprosthesis for supra-aortic branches. In two cases, the stent graft was placed distal to the LSA and ARSA origin. But, in four patients the ARSA was covered. They report that one patient suffered right upper limb weakness, which resolved without further treatment. They argued that preservation of one subclavian artery, preferably the one with the dominant vertebral artery, is enough and can be recommended. Recent reports supported this point of view, finding that selective LSA coverage did not increase the risk of spinal cord ischemia and cerebrovascular accident. 42 Indeed, the RSA might not be as important for perfusion of the spinal cord, and therefore, revascularization may not be necessary to prevent paraplegia. But, most patients are right-handed and rely on a well-perfused arm to maintain their quality of life. It might not be necessary to immediately revascularize the ARSA. But, the reported incidence of five transient right arm ischemic episodes in our review does not support liberal coverage of the ARSA. Therefore, we recommend that in cases of planned coverage of the ARSA ostium adequate revascularization always be considered.

There are several techniques for revascularization of the subclavian arteries. If performed in a hybrid approach, this is done via surgical bypass or transposition. Only one case was reported where the RSA was transposed into the RCCA. 33 All other patients received prosthetic bypasses to the supra-aortic branches. The choice between transposition and bypass should be made according to the anatomical conditions and the operator's preferences. In our case, as well as in earlier reports, 5 the RSA in these cases is often found to be more centrally located than usual. Therefore, transposition is sometimes more difficult. None of the patients received a venous interposition, likely fearing compression by surrounding muscular tissue. For cervical debranching, there are some aspects that have to be considered. First of all, clamping of the carotid artery is always associated with a risk of stroke. This risk is further elevated in cases, where both subclavians are revascularized. Also, the same applies for double cervical incisions, which may potentially involve the risk of dysphonia and diaphragm paralysis. Therefore, some authors recommended staged procedures. 40 Nevertheless, in our literature review, none of these complications were reported.

For total endovascular repair, also a variety of approaches to maintain flow to the supra-aortic branches are available—chimney, periscope/snorkel techniques, or fenestrated devices. Theoretically, chimney or periscope techniques bear the risk of endoleaks, due to the potential avenue that is created between the stents. 9 In the reviewed literature and in similar case series, the authors reported relevant rates of type Ia endoleaks. But, most of these resolved spontaneously within the first month. 9 43 Another question regards whether stent grafts in the ARSA might increase the risk of esophageal compression due to the close proximity of these anatomical structures. This could lead to dysphagia or esophageal perforation with fistula formation. Also, occlusion devices in the proximal ARSA, like coils and plugs, may bear this risk. 37 In our review, with limited follow-up data, no such symptoms were reported for these cases.

Fenestrated and branched endografts are potential alternatives to the chimney and periscope techniques. However, there are limitations, such as manufacturing lead time and high cost. Physician-modified fenestrated stent graft represents an alternate option, especially suitable for high-risk patients in emergency situations. In our literature review, only one case has been successfully treated this way. 9 Off-the-shelf devices are going to be available in the near future and will have to prove their value in emergency cases. So far, only one case has been reported, where a Type B AD with ARSA has been treated with a branched aortic stent graft. 20

Complications during follow-up of these patients were rare. But, follow-up data were actually insufficient, with a mean of only 25 months. For open repair without TEVAR, secondary dilatation of the descending aorta is a relevant issue. For hybrid and total endovascular repair, the incidence of endoleaks is quite an issue, even though most (type I and II) endoleaks spontaneously resolved. Retrograde Type A AD was not observed immediately after implantation, but one case was reported 3 months later. 8 Therefore, a close follow-up of these cases is mandatory. If occlusion devices or stent grafts are deployed in the ARSA, it has to be kept in mind that these may compress the esophagus, leading to dysphagia or even to a fistula. 37 Under what circumstances this happens is still unclear. This may depend on the size of the device or its location. Interestingly, we did find a single report where the aortic stent graft itself led to the compression of the esophagus and fistula formation with a lethal outcome 42 months after implantation. 17 Considering these reported events, lifelong follow-up of these patients is urgently recommended.

There are certain limitations to our study. Even though we were able to form equally distributed treatment groups, the informative value is still limited due to the rare occurrence of this pathology, the retrospective nature, and heterogeneous reporting of the collected data. Unfortunately, we are unable to draw conclusions regarding the durability of the different types of repair, since most reports describe only short or mid-term results. But, long-term results would be of great interest, especially since patients presenting with Type B AD and ARSA are usually younger than the average Type B AD patient from the International Registry of Acute Aortic Dissections (mean age: 53.7 ± 10.4 vs. 63.6 ± 14.1 years, respectively). 44 Also, our analysis might possibly be biased, since successful cases are more likely to be reported than negative results. Therefore, it would be of great help, if ongoing larger registries would provide a more detailed analysis of patients presenting with AD in the presence of an ARSA in a prospective manner. This would aid in comparing outcomes of different treatment strategies and contribute to decision-making in the future.

## Conclusion

In this first-ever detailed analysis of the available literature regarding the treatment of Type B AD in the coexistence of an ARSA, all therapeutic options show excellent results. Nowadays, the open surgical treatment seems to be reserved for younger, male patients often presenting with chronic dissections and larger aortic diameters that may be unsuitable for endovascular treatment. Hybrid and total endovascular therapies are the preferred approaches, especially in emergency situations. All therapies carry potential risks in the long term, but long-time follow-up data are sparse. Therefore, reports of cases with longer follow-up would be desirable to prove their durability.
