# Supplementary material for: How to Treat Type B Aortic Dissections in the Presence of an Aberrant Right Subclavian Artery: A Systematic Review
Source: Aorta (Stamford). 2023 Feb 27;11(1):20–8. doi: 10.1055/s-0042-1757948 (PMC9970753; doi:10.1055/s-0042-1757948)
Supplement: Supplementary file 1 — Supplementary Material [file 10-1055-s-0042-1757948-s210046.pdf]

Supplementary Appendix

Section and Topic	Item no.	Checklist item	Location where item is reported
Title			
Title	1	Identify the report as a systematic review	Page 0
Abstract			
Abstract	2	See the PRISMA 2020 for Abstracts checklist	Page 2
Introduction			
Rationale	3	Describe the rationale for the review in the context of existing knowledge	Page 3
Objectives	4	Provide an explicit statement of the objective(s) or question(s) the review addresses	Page 3
Methods			
Eligibility criteria	5	Specify the inclusion and exclusion criteria for the review and how studies were grouped for the syntheses	Pages 4 and 5
Information sources	6	Specify all databases, registers, Web sites, organisations, reference lists and other sources searched or consulted to identify studies. Specify the date when each source was last searched or consulted	Page 4
Search strategy	7	Present the full search strategies for all databases, registers and Web sites, including any filters and limits used	Suppl. 1
Selection process	8	Specify the methods used to decide whether a study met the inclusion criteria of the review, including how many reviewers screened each record and each report retrieved, whether they worked independently, and if applicable, details of automation tools used in the process	Pages 4 and 5
Data collection process	9	Specify the methods used to collect data from reports, including how many reviewers collected data from each report, whether they worked independently, any processes for obtaining or confirming data from study investigators, and if applicable, details of automation tools used in the process	Pages 4 and 5
Data items	10a	List and define all outcomes for which data were sought. Specify whether all results that were compatible with each outcome domain in each study were sought (e.g., for all measures, time points, analyses), and if not, the methods used to decide which results to collect	Page 5
	10b	List and define all other variables for which data were sought (e.g., participant and intervention characteristics, funding sources). Describe any assumptions made about any missing or unclear information	Suppl. 3
Study risk of bias assessment	11	Specify the methods used to assess risk of bias in the included studies, including details of the tool(s) used, how many reviewers assessed each study and whether they worked independently, and if applicable, details of automation tools used in the process	Page 4
Effect measures	12	Specify for each outcome the effect measure(s) (e.g., risk ratio, mean difference) used in the synthesis or presentation of results	Pages 4 and 5

 © 2023. The Author(s).

(*Continued*)

Section and Topic	Item no.	Checklist item	Location where item is reported
Synthesis methods	13a	Describe the processes used to decide which studies were eligible for each synthesis (e.g., tabulating the study intervention characteristics and comparing against the planned groups for each synthesis [item no. 5])	Page 5
	13b	Describe any methods required to prepare the data for presentation or synthesis, such as handling of missing summary statistics, or data conversions	Page 5
	13c	Describe any methods used to tabulate or visually display results of individual studies and syntheses	Page 5
	13d	Describe any methods used to synthesize results and provide a rationale for the choice(s). If meta-analysis was performed, describe the model(s), method(s) to identify the presence and extent of statistical heterogeneity, and software package(s) used	Page 5
	13e	Describe any methods used to explore possible causes of heterogeneity among study results (e.g., subgroup analysis, metaregression)	Page 5
	13f	Describe any sensitivity analyses conducted to assess robustness of the synthesized results.	Page 5
Reporting bias assessment	14	Describe any methods used to assess risk of bias due to missing results in a synthesis (arising from reporting biases)	Pages 4 and 5
Certainty assessment	15	Describe any methods used to assess certainty (or confidence) in the body of evidence for an outcome	Page 5
Results			
Study selection	16a	Describe the results of the search and selection process, from the number of records identified in the search to the number of studies included in the review, ideally using a flow diagram	Page 6 and ►**Fig. 2**
	16b	Cite studies that might appear to meet the inclusion criteria, but which were excluded, and explain why they were excluded	Suppl. 2
Study characteristics	17	Cite each included study and present its characteristics	Page 6 and Suppl. 3
Risk of bias in studies	18	Present assessments of risk of bias for each included study	Page 5 and N/A
Results of individual studies	19	For all outcomes, present, for each study: (a) summary statistics for each group (where appropriate) and (b) an effect estimate and its precision (e.g., confidence/credible interval), ideally using structured tables or plots	Pages 6 and 7 and ►**Tables 1** and **2**
Results of syntheses	20a	For each synthesis, briefly summarize the characteristics and risk of bias among contributing studies	Page 5 and N/A
	20b	Present results of all statistical syntheses conducted. If meta-analysis was done, present for each the summary estimate and its precision (e.g., confidence/credible interval) and measures of statistical heterogeneity. If comparing groups, describe the direction of the effect	Pages 6 and 7 and ►**Tables 1** and **2**
	20c	Present results of all investigations of possible causes of heterogeneity among study results	Pages 6 and 7 and ►**Tables 1 & 2**
	20d	Present results of all sensitivity analyses conducted to assess the robustness of the synthesized results	Pages 6 and 7 and ►**Tables 1 & 2**

(*Continued*)

 © 2023. The Author(s).

(*Continued*)

Section and Topic	Item no.	Checklist item	Location where item is reported
Reporting biases	21	Present assessments of risk of bias due to missing results (arising from reporting biases) for each synthesis assessed	Page 5 and N/A
Certainty of evidence	22	Present assessments of certainty (or confidence) in the body of evidence for each outcome assessed	Page 5 and N/A
Discussion			
Discussion	23a	Provide a general interpretation of the results in the context of other evidence	
	23b	Discuss any limitations of the evidence included in the review	Pages 12 and 13
	23c	Discuss any limitations of the review processes used	Page 12 and 13
	23d	Discuss implications of the results for practice, policy, and future research	Page 13
Other information			
Registration and protocol	24a	Provide registration information for the review, including register name and registration number, or state that the review was not registered	Page 4
	24b	Indicate where the review protocol can be accessed, or state that a protocol was not prepared	Page 4
	24c	Describe and explain any amendments to information provided at registration or in the protocol	N/A
Support	25	Describe sources of financial or non-financial support for the review, and the role of the funders or sponsors in the review	Page 1
Competing interests	26	Declare any competing interests of review authors	Page 0
Availability of data, code and other materials	27	Report which of the following are publicly available and where they can be found: template data collection forms; data extracted from included studies; data used for all analyses; analytic code; any other materials used in the review	Suppl. 3

From: Page MJ, McKenzie JE, Bossuyt PM, Boutron I, Hoffmann TC, Mulrow CD, et al. The PRISMA 2020 statement: an updated guideline for reporting systematic reviews. BMJ 2021;372:n71.
For more information, visit: *http://www.prisma-statement.org/*.

Supplementary Material S1

Literature Search Strategy

Component 1: aortic dissection and aberrant right subclavian artery
Search query: ((aberrant right subclavian artery) AND (“aortic dissection”[All Fields])) AND ((“2000”[Date - Publication]: “3000”[Date - Publication]))
Component 2: Type B Aortic Dissection and Aberrant right subclavian artery
Search query: ((aberrant right subclavian artery) AND (“type B aortic dissection”[All Fields])) AND ((“2000”[Date - Publication]: “3000”[Date - Publication]))
Date of query: February 1, 2021.

 © 2023. The Author(s).

Supplementary Material S2

References of all Excluded Studies with Reason for Exclusion

Exclusion Criterium: Type A Aortic Dissection

1. Li JR, Ma WG, Chen Y, et al. Total arch replacement and frozen elephant trunk for aortic dissection in aberrant right subclavian artery. Eur J Cardiothorac Surg 2020;58(1):104–111 PubMed
2. Li JR, Ma WG, Zheng J, et al. [Surgical treatment for type Stanford A aortic dissection with Kommerell's diverticulum]. Zhonghua Wai Ke Za Zhi 2020;58(2):137–141 PubMed
3. Kim JH, Song KS, Kim JB. Open repair of thoracoabdominal aortic aneurysm in a 46-year-old man with pleural adhesions and aberrant right subclavian artery. Tex Heart Inst J 2018;45(3):179–181 PubMed
4. Liu NN, Sun LZ. [Progress of problems related to the reconstruction of aortic arch in acute type A aortic dissection]. Zhonghua Wai Ke Za Zhi 2017;55(4):311–315 PubMed
5. Tanaka Y, Kitamura T, Horai T, Miyaji K. Two-stage operation for Stanford type A acute aortic dissection originating from Kommerell's diverticulum. Interact Cardiovasc Thorac Surg 2016;22(5):695–697 PubMed
6. Omura A, Yoshida M, Matsuda H, Mukohara N. Acute type A aortic dissection in a patient with right aortic arch and an aberrant left subclavian artery arising from Kommerell diverticulum. Interact Cardiovasc Thorac Surg 2016;22(4):493–494 PubMed
7. Ren CW, Lai YQ, Yang S, Xu SD, Sun LZ. Four-branch prosthetic graft used for stanford type A aortic dissection with aberrant right subclavian artery. Chin Med J (Engl) 2015;128(11):1558 PubMed
8. Ren C, Sun L, Huang L, Lai Y, Yang S, Xu S. Hybrid procedure for acute stanford type a aortic dissection with aberrant right subclavian artery. J Card Surg 2015;30(3):274–275 PubMed
9. Li C, Shi J, Shi Y, Guo Y. Unusual association of type A aortic dissection and aberrant right subclavian artery in Marfan syndrome. Int J Cardiol 2014;175(1):e14–e15 PubMed
10. Tanaka K, Tanaka K, Natsume K, Yamamoto K, Hiraiwa T. Successful surgical exclusion of rapidly expanding kommerell diverticulum following a total arch replacement for an acute type a aortic dissection. Ann Vasc Dis 2014;7(3):339–342 PubMed
11. Kitamura H, Kimura A, Fukaya S, Okawa Y, Komeda M. Emergent total arch replacement for acute type A aortic dissection with aberrant right subclavian artery in a systemic lupus erythematosus patient. Gen Thorac Cardiovasc Surg 2016;64 (1):25–27 PubMed
12. Battaloglu B, Secici S, Colak C, Disli OM, Erdil N, Kutlu R. Aberrant right subclavian artery and axillary artery cannulation in type a aortic dissection repair. Ann Thorac Surg 2013;96(1):e1–e2 PubMed
13. Guo C, Zhu K, Xu D, Wang C. Open triple-branched stent graft applied to patient of acute type A aortic dissection with aberrant right subclavian artery. J Cardiothorac Surg 2013;8:85 PubMed
14. Vistarini N, Aubert S, Gandjbakhch I, Bonnet N. Aberrant subclavian artery as origin of aortic dissection. Eur J Cardiothorac Surg 2008;34(5):1109 PubMed
15. Kikuchi K, Makuuchi H, Oono M, Murakami H, Suzuki T, Ando T. Surgery for aortic dissection involving an aberrant right subclavian artery. Jpn J Thorac Cardiovasc Surg 2005;53(12):632–634 PubMed
16. Mogi K, Okimoto M, Okada Y. Emergent ascending and arch replacement for acute type A aortic dissection with anomalies of the aortic arch. Gen Thorac Cardiovasc Surg 2016;64(1):25–27 PubMed
17. Misumi T, Kudo M, Ito T, Cho Y, Kumamaru H. Acute aortic dissection involving an aberrant right subclavian artery. Jpn J Thorac Cardiovasc Surg 2002;50(3):119–121 PubMed

Exclusion Criterium: Latrogenic Lesion without Surgical Therapy

1. Wang P, Wang Q, Bai C, Zhou P. Iatrogenic aortic dissection following transradial coronary angiography in a patient with an aberrant right subclavian artery. J Int Med Res 2020;48(8):300060520943789 PubMed
2. Rafiq A, Chutani S, Krim NR. Coronary intervention and arteria lusoria—not simple as it may seem. Catheter Cardiovasc Interv 2019;93(3):563–564 PubMed

Exclusion Criterium: Case Series with Multiple Aberrant Right Subclavian Artery Pathologies: Unable to Differentiate Data from Patients with Type B Aortic Dissection

1. Gray SE, Scali ST, Feezor RJ, et al. Safety and efficacy of a hybrid approach for repair of complicated aberrant subclavian arteries. J Vasc Surg 2020;72(6):1873–1882 PubMed
2. Dzsinich C, Darabos G, Székely L, et al. Kommerell diverticulum—egy ritka betegség ritka szövődményei. Magy Seb 2020;73 (2):61–68 PubMed
3. Wooster M, Back M, Sutzko D, Gaeto H, Armstrong P, Shames M. A 10-year experience using a hybrid endovascular approach to treat aberrant subclavian arterial aneurysms. Ann Vasc Surg 2018;46:60–64 PubMed

 © 2023. The Author(s).

4. Arazińska A, Polguj M, Szymczyk K, Kaczmarska M, Trębiński Ł, Stefańczyk L. Right aortic arch analysis—anatomical variant or serious vascular defect? BMC Cardiovasc Disord 2017;17(1):102 PubMed
5. van Bogerijen GHW, Patel HJ, Eliason JL, et al. Evolution in the management of aberrant subclavian arteries and related Kommerell diverticulum. Ann Thorac Surg 2015;100(1):47–53 PubMed
6. Cinà CS, Althani H, Pasenau J, Abouzahr L. Kommerell's diverticulum and right-sided aortic arch: a cohort study and review of the literature. J Vasc Surg 2004;39(1):131–139 PubMed

Exclusion Criterium: Radiological Description

1. Plotkin A, Ng B, Han SM, et al. Association of aberrant subclavian arteries with aortic pathology and proposed classification system. J Vasc Surg 2020;72(5):1534–1543 PubMed
2. Lami N, Laissy JP, Gibeault M, Feldman L, Schouman-Claeys E. Aortic dissection and aberrant right subclavian artery: CT and MR findings. J Radiol 2002;83(5):653–655
3. Janssen M, Breburda CS, van Geuns RJ, et al. Images in cardiovascular medicine. Aberrant right subclavian artery mimics aortic dissection. Circulation 2000;101(4):459–460 PubMed

Exclusion Criterium: Only Description of This Pathology

1. Ikeno Y, Koide Y, Matsueda T, et al. Anatomical variations of aortic arch vessels in Japanese patients with aortic arch disease. Gen Thorac Cardiovasc Surg 2019;67(2):219–226 PubMed
2. Shalhub S, Schäfer M, Hatsukami TS, et al. Association of variant arch anatomy with type B aortic dissection and hemodynamic mechanisms. J Vasc Surg 2018;68(6):1640–1648 PubMed
3. Uchino A, Uwabe K, Osawa I. Absent right common carotid artery associated with aberrant right subclavian artery. Neuroradiol J 2018;31(3):305–308 PubMed
4. Jones CS, Verde F, Johnson PT, Fishman EK. Nontraumatic subclavian artery abnormalities: spectrum of MDCT findings. AJR Am J Roentgenol 2016;207(2):434–441 PubMed
5. Baek WK, Kim YS, Lee M, Yoon YH, Kim JT, Lim HK. Axillary artery cannulation in acute aortic dissection: a word of caution. Ann Thorac Surg 2016;101(4):1573–1574 PubMed
6. Tanaka A, Milner R, Ota T. Kommerell's diverticulum in the current era: a comprehensive review. Gen Thorac Cardiovasc Surg 2015;63(5):245–259 PubMed
7. Maxwell BG, Harrington KB, Beygui RE, Oakes DA. Congenital anomalies of the aortic arch in acute type-a aortic dissection: implications for monitoring, perfusion strategy, and surgical repair. J Cardiothorac Vasc Anesth 2014;28(3):467–472 PubMed
8. Rasmussen DK, Dougherty J. Aortic dissection with vascular abnormalities. J Am Osteopath Assoc 2011;111(6):407–409
9. Nasir A, Jadoon M, Ellis PK, Graham AN. Kommerell's diverticulum, risk factor for aortic dissection. J Card Surg 2009;24(4):463 PubMed
10. Dhareshwar J, Estrera AL, Porat EE, Azizzadeh A, Safi HJ. Acute type B dissection with involvement of an aberrant right subclavian artery: an unusual presentation and a diagnostic challenge. J Thorac Cardiovasc Surg 2006;132(3):689 PubMed

Exclusion Criterium: No Treatment

1. Chowdhury Y, Shaikh SA, Salman A, Marmur JD, McFarlane IM. Aberrant right subclavian artery and stanford type B aortic dissection. Am J Med Case Rep 2020;8(8):247–249 PubMed
2. Horrill T. Acute aortic dissection following treatment for castration-resistant prostate cancer. Oncol Nurs Forum 2016;43(4):413–416 PubMed
3. Singh S, Grewal PD, Symons J, Ahmed A, Khosla S, Arora R. Adult-onset dysphagia lusoria secondary to a dissecting aberrant right subclavian artery associated with type B acute aortic dissection. Can J Cardiol 2008;24(1):63–65 PubMed

Exclusion Criterium: Comment

1. Bozzani A, Arici V, Ragni F, Odero A. Comment on aortic dissection in the presence of an aberrant right subclavian artery. Ann Vasc Surg 2013;27(3):396–397 PubMed
2. Exclusion Criterium: Aberrant Left Subclavian Artery not Aberrant Right Subclavian Artery
3. Zhang M, Yuan Y, Hu Y, et al. Endovascular repair with the chimney technique for stanford type B aortic dissection involving right-sided arch with aberrant left subclavian artery. Ann Vasc Surg 2014;28(7):1798.e7–1798.e10 PubMed
4. Hsu HL, Huang CY, Chen JS. Total endovascular repair for acute type B dissection in the setting of right aortic arch with aberrant left subclavian artery and Kommerell diverticulum. J Thorac Cardiovasc Surg 2015;150(2):409–411 PubMed
5. Guo H, Sun X, Yu C, Shu C. A case report of frozen elephant trunk combined with endovascular treatment for acute aortic dissection of Kommerell's diverticulum involving right aortic arch and descending aorta. Medicine (Baltimore) 2018;97(15):e0166 PubMed

Exclusion Criterium: No Dissection

1. Tanaka H, Hamada M, Higuchi S, Itoh T. Total arch replacement through a mediansternotomy for an aortic arch aneurysm with an aberrant right subclavian artery [in Japanese]. Kyobu Geka 2011;64(13):1158–1161 PubMed
2. Sefránek V, Vulev I, Slysko R, Klepanec A, Zita Z, Balázs T. Successfully combined management of the abberant retroesophageal arteria subcalvia dextra aneurysm (arteria lusoria) [in Slovak]. Rozhl Chir 2010;89(1):64–68 PubMed

 © 2023. The Author(s).

Supplementary Material S3 Total endovascular procedures

Study and year (country)	Number of patients	Age (y)	Male (%)	Symptoms	Dissected ARSA	Maximum aortic diameter (mm)	Procedure	Hospital stay (d)	In-hospital mortality	Major complications (death, stroke)	Minor complications	Follow-up complications	Follow-up (no)
Li et al and 2007 (China)	1	47	100	Persistent pain	Yes	NR	TEVAR	NR	0	0	0	0	3
Samura et al and 2014 (Japan)	1	72	0	Persistent pain	Yes	NR	TEVAR + chimney	NR	0	0	0	0	3
Mosquera et al and 2007 (Spain)	1	80	100	Persistent pain	No	46	TEVAR	6	0	0	0	NR	0
Ding et al and 2017 (China)	1	70	100	Persistent pain	No	NR	TEVAR + snorkel	7	0	0	0	0	6
Odero et al and 2007 (Italy)	1	59	100	Persistent pain	Yes	NR	TEVAR	NR	0	0	Transient arm ischemia	0	60
Zhang et al and 2019 (China)	15	55	87	Persistent pain 7/15<br>Pleural effusion 2/15<br>Left femoral artery malperfusion 1/15<br>Refractory hypertension 2/15	1/15	36.91	FEVAR: 1/15<br>TEVAR: 6/15<br>TEVAR + snorkel: 8/15	12	0	1	Transient arm ischemia 2/15<br>Endoleak Ia 1/15	0	33
Zhou et al and 2017 (China)	13	58	61.5	11/13 (Persistent pain, refractory hypertension, visceral ischemia, rupture, pleural effusion, rapid progression)	NR	40.3	TEVAR: 6/13<br>TEVAR + chimney: 3/13<br>TEVAR + snorkel: 4/13	NR	1	1	Endoleak Ia 1/13<br>Transient arm ischemia 1/13	Fatal MI 2/12<br>Stroke 1/12<br>Endoleak Ib 1/12	36
Verzini et al and 2014 (Italy)	1	67	0	Persistent pain	No	60	TEVAR	NR	0	0	0	Death (aorto-esophageal fistula)	42
Baccin et al and 2005 (Brasil)	1	42	100	Persistent pain	No	NR	TEVAR	NR	NR	NR	NR	NR	0
Elghoneimy et al and 2019 (Saudi Arabia)	1	51	100	Persistent pain	No	NR	TEVAR	3	0	0	0	0	6
Sun et al and 2018 (China)	1	57	100	Persistent pain	NR	NR	BEVAR	NR	0	0	0	0	12
Kawatani et al and 2015 (Japan)	1	71	100	Persistent pain	No	NR	TEVAR	14	0	0	0	distal PAU	1

Abbreviations: ARSA, aberrant right subclavian artery; BEVAR, branched endovascular aortic repair; FEVAR, fenestrated endovascular aortic repair; MI, myocardial infarction; NR, not reported; PAU, penetrating aortic ulcer; TEVAR, thoracic endovascular aortic repair.

 © 2023. The Author(s).

Supplementary Material S4 Hybrid procedures

Author and year (country)	Number of patients	Age (y)	Male (%)	Symptoms	Dissected ARSA	Maximum aortic diameter (mm)	Procedure	Hospital stay (d)	In-hospital mortality	Major complications (death, stroke)	Minor complications	Follow-up complications	Follow-up (mo)
Floris Vos et al and 2002 (the Netherlands)	1	59	100	Persistent pain	No	NR	TEVAR + Debranching RSA in RCCA	7	0	0	0	0	19
Hamidian-Jahromi et al and 2013 (the United States)	1	41	0	Persistent pain	Yes	NR	TEVAR + LCCA-LSA-Bypass + coiling ARSA	8	0	0	0	0	12
Cooper et al and 2009 (the United Kingdom)	1	44	0	Dyspnea	Yes	65	Ascendo-RCCA-RSA Bypass + TEVAR	5	0	0	0	0	3
Guzman et al and 2012 (the United States)	2	64	100	Persistent pain	No	NR	TEVAR + RCCA-RSA-Bypass	NR	0	0	SMA Stenosis:1/2	Endoleak II	11
Huanyu Ding et al and 2018 (China)	16	51.3	75	16/16 (Persistent pain, refractory hypertension, visceral or renal ischemia, true lumen collapse, rapid progression)	5/16	36	TEVAR + Bypass: LCCA-LSA + RCCA-RSA 5/16<br>LCCA-LSA-RSA 5/16<br>RCCA-LCCA-RSA-LSA 4/16<br>RCCA-RSA 2/16	NR	0	0	Endoleak Ia: 3/16<br>Endoleak II: 1/16<br>Plexus injury: 2/16	Bypass occlusions: 2/16<br>Retrograde Type A AD: 1/16	30.9
Kwok et al and 2008 (China)	1	64	100	nr	No	NR	TEVAR + Bypass (RCCA-LSA)	NR	0	0	0	0	1
Son et al and 2018 (Korea)	1	32	0	Persistent pain	No	NR	TEVAR + RCCA-RSA-Bypass	6	0	0	0	NR	0
Quinones-Aldrich et al and 2013 (United States)	1	44	0	None	No	60	Ascendo-RCCA-LCCA + RCCA-RSA + LCCA-LSA Bypass + TEVAR	NR	0	0	0	0	24
Chan et al and 2007 (UK)	1	70	100	Persistent pain	No	55	TEVAR + RCCA-LCCA + RCCA-RSA + LCCA-LSA Bypass	5	0	0	0	Fatal MI after 2 months	2

Abbreviations: AD, aortic dissection; ARSA, aberrant right subclavian artery; LCCA, left carotid artery; LSA, left subclavian artery; MI, myocardial infarction; NR, not reported; PAU, penetrating aortic ulcer; RCA, right carotid artery; RCCA, right common carotid artery; RSA, right subclavian artery; SMA, superior mesenteric artery; TEVAR, thoracic endovascular aortic repair.

 © 2023. The Author(s).

Supplementary Material S5 Open surgical procedures

Author and year (country)	Number of patients	Age (y)	Male (%)	Symptoms	Dissected ARSA	Maximum aortic diameter (mm)	Procedure	Hospital stay (d)	In-hospital mortality	Major complications (death, stroke)	Minor complications	Follow-up complications	Follow-up (mo)
Guzman et al and 2012 (the United States)	1	49	100	None	Yes	46	FET	NR	0	1	0	0	24
Murana et al and 2020 (Italy)	1	55	0	Persistent pain	Yes	39	FET	28	0	0	Prolonged weaning	NR	0
Di Stefano et al and 2014 (Italy)	1	39	100	Persistent pain	NR	NR	FET	NR	0	0	NR	NR	0
Belluschi et a and 2020 (Italy)	1	61	100	True lumen collapse	Yes	NR	FET	15	0	0	Bleeding	0	3
Zhu et al and 2016 (China)	7	48	100	None 7/7	No	46.4	TAR + TEVAR	NR	0	0	Prolonged weaning 1/7	0	31
Abuharb et al and 2019 (China)	1	54	100	Persistent pain	Yes	NR	FET	13	0	0	0	0	1
Huang et al 2019 (China)	1	39	0	Persistent pain	No	NR	FET	NR	0	0	0	0	16
Ghanem et al and 2016 (France)	1	53	100	Persistent pain	Yes	NR	FET	7	0	0	0	0	3
Yanagihara et al and 2015 (Japan)	1	43	100	Persistent pain	No	56	distal arch repair	9	0	0	0	NR	0
Chang et al and 2020 (China)	4	47.8	100	Persistent pain 2/4	3/4	NR	TAR: 3/4<br>FET: 1/4	NR	1	1	Compression of trachea 1/4 critical illness poly-neuropathy 1/4	0	20
Ikeno et al and 2019 (Japan)	1	77	100	Persistent pain	NR	50.9	TAR	NR	0	0	0	0	204
Kwon et al and 2020 (Korea)	2	41.5	100	Arm ischemia 1/2	NR	67.5	TAR	NR	0	1	Temporary paraparesis and wound infection with mediastinitis 1/2	Secondary TAAA	30

Abbreviations: FET, frozen elephant trunk; NR, not reported; TAAA, thoracoabdominal aneurysm; TAR, total arch repair.
